# Neural Basis of Body Image: How to Lose Inches at the (Perceived) Flick of the Wrist

**DOI:** 10.1371/journal.pbio.0030439

**Published:** 2005-11-29

Wouldn't it be nice if you could change your body image by placing a vibrating gadget on your wrist? As it happens, you can—though under controlled circumstances. Vibrating skin over the tendon of a joint extensor muscle triggers the vivid sensation that the joint is passively flexing, even though it's not. When the hand is touching the waist, nose, or some other body part, a person can feel the wrist bending and the body part stretching or shrinking—in what's aptly called the Pinocchio illusion.

Vibrations on the skin over a muscle tendon cause the perceptual illusion by exciting sensory nerve endings in the tendon that send signals to brain areas that process touch and motor control, the primary somatosensory cortex and the primary motor cortex. The somatosensory cortex creates neural maps of the body surface, and receives sensory inputs from receptors in the peripheral nervous system. But these peripheral receptors carry no information about the relative size of body parts, and the brain has no specialized neurons to receive such information. The neural map of body size and shape are likely represented in a relative way by integrating signals from the relevant body parts and visual cues. The parietal lobes may play a role, based on reports that patients with parietal cortex injuries imagine changes in the size and shape of various body parts. Still, it's not clear how the brain integrates the relevant information to compute body image. [Fig pbio-0030439-g001]

**Figure pbio-0030439-g001:**
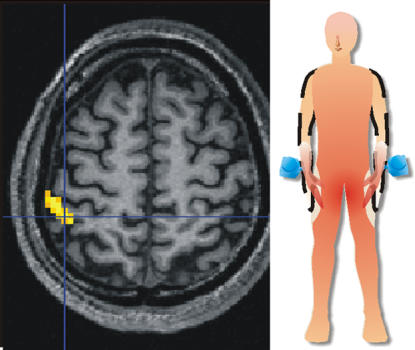
Higher-order somatosensory areas in the parietal cortex mediate perceived changes in body shape and size

To investigate the neural correlates of body image, H. Henrik Ehrsson, Eiichi Naito, and their colleagues recruited 24 participants to model the “waist shrinking illusion,” and then scanned their brains with functional magnetic resonance imaging (fMRI). The authors hypothesized that higher-order somatosensory areas in the parietal cortex would reflect the perceived changes in waist size, and designed the study to isolate illusion-linked brain activity by varying participants' hand position (body contact/no contact, or free) and the vibration site (tendon/skin, or beside the tendon).

After participants experienced each possible combination of hand position and vibration site, they answered “now” when they felt the illusion, and then chose a picture from six different body configurations that best represented their experience. They rated the vividness of the experience, on a scale of zero to nine (absolutely realistic), and then moved their wrists to show what they felt so the authors could measure the angle. At the same time, electromyograms (EMGs) recorded muscle stimulation.

Seven participants did not reliably experience the illusion and so were not scanned. The other 17 participants underwent six experimental trials (two baselines, with hands resting, were added) while their brains were scanned (while lying in the fMRI machine). In three trials, participants' hands lay freely, but supported, by their side without touching the body (tendon free/skin free/rest free). In the other three trials, the palms of the hand were in direct contact with their sides (tendon contact/skin contact/skin free), while a strap allowed them to relax their arms.

During the tendon contact condition, all 17 participants sensed their hands flexing and their waist shrinking. The degree of flexion corresponded to a 28% waist shrinkage. This sensation was vivid, reliable, and quick to start. The EMGs showed no muscle activity in over 70% of the participants, and muscle activity wasn't significantly different in tendon contact and tendon free, confirming that muscle stimulation did not account for the illusion. The brain regions showing most activity during the illusory perception were in the left parietal lobe, within the anterior intraparietal sulcus (a sulcus is an inward fold of the brain) and extending toward the postcentral sulcus.

Participants who reported the strongest shrinking waist illusion also showed the strongest activity in the postcentral sulcus and the anterior left intraparietal cortex—activity that was not observed in participants who felt illusory wrist movement when their hands were not touching their body—confirming a link between these brain regions and the shrinking waist illusion. When the brain receives conflicting sensory information from the vibrated wrists and the sensory inputs of the hands on the waist, the brain recalibrates the relative size of the wrist and shape of the waist, creating the illusion that the waist is shrinking as the hands are bending inward. Altogether, these results suggest that the brain computes body image by integrating signals from the skin, joints, and muscles through hierarchical processing in the somatosensory system. The researchers could elicit this illusion as many times as needed for the fMRI experiment, but there's no indication that a portable device will hit the consumer market anytime soon. —*Liza Gross*

